# A PQS-Cleaving Quorum Quenching Enzyme Targets Extracellular Membrane Vesicles of *Pseudomonas aeruginosa*

**DOI:** 10.3390/biom12111656

**Published:** 2022-11-08

**Authors:** Alba Arranz San Martín, Steffen Lorenz Drees, Susanne Fetzner

**Affiliations:** Institute of Molecular Microbiology and Biotechnology, University of Münster, Corrensstraße 3, 48149 Münster, Germany

**Keywords:** dioxygenase, *Pseudomonas* quinolone signal, *Pseudomonas aeruginosa*, quorum quenching enzyme, outer membrane vesicles, membrane-protein interaction, homo-FRET

## Abstract

The opportunistic pathogen *Pseudomonas aeruginosa* uses quorum sensing to control its virulence. One of its major signal molecules, the *Pseudomonas* quinolone signal PQS, has high affinity to membranes and is known to be trafficked mainly via outer membrane vesicles (OMVs). We previously reported that several 3-hydroxy-4(1*H*)-quinolone 2,4-dioxygenases (HQDs) catalyze the cleavage of PQS and thus act as quorum quenching enzymes. Further analysis showed that, in contrast to other HQDs, the activity of HQD from *Streptomyces bingchenggensis* (HQD*_S_._b_.*) was unexpectedly stabilized by culture supernatants of *P. aeruginosa*. Interestingly, the stabilizing effect was higher with supernatants from the strain PA14 than with supernatants from the strain PAO1. Heat treatment and lyophilization hardly affected the stabilizing effect; however, fractionation of the supernatant excluded small molecules as stabilizing agents. In a pull-down assay, HQD*_S_._b_.* appeared to interact with several *P. aeruginosa* proteins previously found in the OMV proteome. This prompted us to probe the physical interaction of HQD*_S_._b_.* with prepared extracellular membrane vesicles. Homo-FRET of fluorescently labeled HQD*_S_._b_.* indeed indicated a spatial clustering of the protein on the vesicles. Binding of a PQS-cleaving enzyme to the OMVs of *P. aeruginosa* may enhance PQS degradation and is highly reconcilable with its function as a quorum quenching enzyme.

## 1. Introduction

Bacteria employ cell-to-cell communication to sense and coordinatively respond to the state or activity of their population. This phenomenon, known as quorum sensing (QS), relies on the production of small diffusible molecules, termed autoinducers, that accumulate in the extracellular milieu as the cell density increases. Numerous physiological processes and collective behaviors including bioluminescence, sporulation, biofilm formation, antibiotic resistance and the production of multiple virulence factors are under the regulation of QS mechanisms (reviewed in [1,2]).

QS signal molecules are chemically diverse, and many bacterial species produce several signal molecules that are integrated into interconnected regulatory networks. For instance, the QS network of the opportunistic pathogen *Pseudomonas aeruginosa* comprises three interdependent and overlapping systems, two depending on *N*-acylhomoserine lactones (AHL), and another system relying on 2-alkyl-4(1*H*)-quinolones (AQ) as QS signal molecules (reviewed in [3]). Within the *pqs* system, 2-heptyl-3-hydroxy-4(1*H*)-quinolone, also known as the *Pseudomonas* quinolone signal (PQS), and its biosynthetic precursor 2-heptyl-4(1*H*)-quinolone (HHQ) act as autoinducers and are involved in the regulation of several virulence factors such as the siderophore pyoverdine, rhamnolipid biosurfactants, elastase and the redox-active pigment pyocyanin [4,5,6]. In addition to its signaling properties, PQS mediates iron acquisition and cytotoxicity against competing microbes, chelates ferric iron and modulates host immune responses. Due to its hydrophobic nature, PQS preferentially localizes into membranes. Its incorporation into the outer membrane even induces the formation of outer membrane vesicles (OMVs), which encase PQS and traffic it within the population [7].

As carriers of cargo such as virulence factors, toxins and nucleic acids, OMVs produced by Gram-negative bacteria play a pivotal role in bacterial pathogenesis and in promoting bacterial survival under stress conditions (reviewed in [8,9]). *P. aeruginosa* packages small molecules and an assortment of proteins into OMVs, including PQS, the virulence-related enzymes alkaline phosphatase, hemolytic phospholipase C, β-lactamase, hemolysin, pro-elastase and the cystic fibrosis transmembrane conductance regulator (CFTR) inhibitory factor Cif [10,11,12].

Due to the involvement of QS in bacterial pathogenesis, interference with QS circuits represents an attractive anti-virulence strategy [13]. Interference with QS, or quorum quenching (QQ), can be achieved by targeting signal biosynthesis, the signal receptor or the signal itself. With respect to the latter, numerous QQ enzymes acting on AHLs have been described, whereas only a small number of enzymes are known to inactivate other autoinducer classes [14].

Recently, we described the protein family of 3-hydroxy-4(1*H*)-quinolone 2,4-dioxygenases (HQDs), which includes members that catalyze the cleavage of PQS into carbon monoxide and *N*-octanoylanthranilic acid [15]. Among these, the dioxygenase HodC in a previous study was shown to attenuate the production of the virulence factors lectin A, pyocyanin and rhamnolipids when exogenously added to *P. aeruginosa* cultures, despite its low activity towards PQS and its susceptibility towards *P. aeruginosa* exoproteases [16]. The HQD family also includes the dioxygenases AqdC from *Mycobacterium abscessus* subsp. *abscessus*, AqdC1 and AqdC2 from *Rhodococcus erythropolis*, and HQD*_S_._b_.* from *Streptomyces bingchenggensis* that have preferential activity towards PQS as a substrate [15,17,18]. The addition of HQD*_S_._b_.* to *P. aeruginosa* cultures significantly quenched AQ levels and attenuated the production of pyocyanin and pyoverdine; moreover, the pre-incubation of *P. aeruginosa* PAO1 with this enzyme increased the survival rate of *Galleria mellonella* larvae when infected with this strain [19]. During our studies, and much to our surprise, we observed a pronounced stabilization of the HQD*_S_._b_.* enzyme by cultures or culture supernatant of *P. aeruginosa*. The present study aimed to identify the stabilizing agent and led to the discovery of a potentially targeted mechanism by which binding to extracellular membrane vesicles stabilizes the quorum quenching enzyme and likely enhances the degradation of PQS quorum sensing signal molecules.

## 2. Materials and Methods

### 2.1. Materials

Lipopolysaccharide (LPS) from *P. aeruginosa* 10 and PQS were purchased from Sigma Aldrich (Schnelldorf, Germany).

### 2.2. Bacterial Strains

The *P. aeruginosa* strains PA14 [20], PAO1 (Nottingham strain) and PAO1-D4, a transposon mutant carrying an insertion in the exopolysaccharide biosynthesis gene *pslJ* (PA2240) [21], were used in this study. *Escherichia coli* BL21(DE3) was used for the heterologous production of proteins.

Heterologous production and purification of PQS dioxygenases: Recombinant *E. coli* BL21(DE3) strains harboring pET23a::*hodC* [22], pET28b(+)::*his_8_-aqdC* [18] or pET28b(+)::*his_8_-hqd_S_._b_*. [15] were grown aerobically at 37 °C in LB with appropriate antibiotics. The cultures were supplemented with 0.25 mM IPTG at an OD_600_ of 0.5–0.8 and incubated for approximately 16 h at 15 °C overnight. The cells were harvested by centrifugation (12,000× *g*, 30 min, 4 °C), resuspended in lysis buffer (20 mM Tris-HCl, pH 8.0, 300 mM NaCl, 0.05% NP-40) and disrupted by sonication. Cell debris was removed by centrifugation (38,000× *g*, 40 min, 4 °C), and the supernatant was filtered. Proteins were purified to electrophoretic homogeneity by Ni^2+^-nitrilotriacetate (Ni-NTA) affinity chromatography and stored in a buffer containing 20 mM Tris-HCl, pH 8.0 and 10% glycerol at −80 °C until further use.

### 2.3. Enzyme Activity Assay and Protein Determination

The activity of PQS dioxygenases was determined at 30 °C in a continuous spectrophotometric assay by measuring PQS consumption at 337 nm. The assay contained 20 µM PQS in buffer (50 mM Tris-HCl, pH 8.0, 2 mM EDTA, 10% (*w*/*v*) polyethylene glycol 1500 and 4% (*v*/*v*) dimethyl sulfoxide) and appropriate amounts of enzyme or enzyme-containing sample. The extinction coefficient of PQS in the assay buffer was 10,169 M^−1^ cm^−1^ at the given conditions. The concentrations of electrophoretically pure PQS dioxygenases were determined spectrophotometrically at 280 nm using the NanoPhotometer^®^ N60 (IMPLEN, Inc., Westlake Village, CA, USA); extinction coefficients were predicted using the ProtParam tool (https://web.expasy.org/protparam/, accessed on 23 October 2020) [23]. The Bradford assay as modified by Zor and Selinger [24], or a commercially available BCA (bicinchoninic acid) assay kit (Sigma), both with bovine serum albumin as the standard protein, were used for other protein-containing samples.

### 2.4. Preparation of P. aeruginosa Culture Supernatants and Incubation with PQS Dioxygenases

*P. aeruginosa* strains were grown in LB or M63 minimal medium [25] (supplemented with 0.2% glucose, 0.1% casaminoacids and 1 mM magnesium sulfate) overnight at 37 °C with shaking at 150 rpm. These pre-cultures were used to inoculate fresh medium to an OD_600_ of 0.045, and the cells were further grown for 8–10 h at 37 °C and 140 rpm. Stationary phase supernatants were collected by centrifugation (13,440× *g*, 15 min, 4 °C) and stored at −80 °C until further use. If required, the cultures were adjusted to the same OD_600_ prior to centrifugation. For the analysis, 500 µL/mL of culture supernatant was incubated in a buffer (20 µM Tris-HCl, pH 8.0) with the enzyme of interest (1 U/mL), and the enzymatic activity was measured at the specified time points.

*P. aeruginosa* PA14 cell-free supernatants were also heated at 95 °C for 20 min or lyophilized. The lyophilized supernatants were then resuspended in sterile distilled H_2_O prior to incubation with PQS dioxygenases.

Fractionation of *P. aeruginosa* PA14 cell-free supernatants was conducted by ultrafiltration using membrane concentrators (VIVASPIN, Sartorius Lab Instruments, Göttingen, Germany) with cut-off sizes of 10, 30 and 50 kDa. Following centrifugation, permeate and retentate fractions were collected to assess their effect on the enzymatic activity of HQD*_S_._b_.*

### 2.5. Pull-Down Assay with PQS Dioxygenases as a Bait

Purified His_8_-tagged HQD*_S_._b_.* and AqdC were incubated with *P. aeruginosa* PA14 cell-free supernatants for one hour at 4 °C, then the mixtures were loaded onto Ni-NTA agarose columns. Alternatively, the purified enzymes were loaded directly onto a Ni-NTA agarose column, followed by the addition of *P. aeruginosa* cell-free supernatant. In both approaches, a washing step with 5 column volumes of buffer (20 mM Tris-HCl, pH 8, 300 mM NaCl, 10 mM imidazole) was applied before eluting AqdC or HQD*_S_._b_.* (with or without co-bound *P. aeruginosa* proteins) using a gradient from 10 to 300 mM imidazole. The collected fractions were concentrated by ultrafiltration, and protein content was estimated. Proteins were separated by SDS-PAGE, and Coomassie-stained gel bands were excised to identify the pulled-down proteins. In-gel tryptic digestion and ESI–MS analysis of the samples was performed by the IZKF Unit Core Proteomics, Münster, Germany.

### 2.6. Preparation of P. aeruginosa Extracellular Membrane Vesicles

Fractions of *P. aeruginosa* supernatants enriched in OMVs were prepared according to Bauman and Kuehn [26]. *P. aeruginosa* PA14 cultures were grown in LB to the stationary phase. Cells were pelleted by centrifugation (12,000× *g*, 15 min, 4 °C), and the supernatant was filter-sterilized through a 0.45 µm PVDF membrane to remove remaining cell debris. The vesicles contained in the supernatant were precipitated with 75% ammonium sulfate at 4 °C for at least 4 h, pelleted (12,000× *g*, 40 min, 4 °C) and dialyzed with HEPES (50 mM, pH 7.5). Then, vesicle samples were concentrated by ultrafiltration (10 kDa cut-off membrane) and adjusted to 45% OptiPrep™ Density Gradient Medium (Sigma-Aldrich, St. Louis, MO, USA) in HEPES (50 mM, pH 7.5). OptiPrep™ media were layered over the 2 mL vesicle sample as follows: 2 mL 40%, 2 mL 35%, 2 mL 30%, 2 mL 25% and 2 mL 20% OptiPrep™/HEPES. The OptiPrep™ gradients were centrifuged (100,000× *g*, 20 h, 4 °C), and 1 mL fractions were removed from the top. Fractions containing vesicles were dialyzed against phosphate-buffered saline (PBS), aliquoted and stored at −80 °C until required. The lipid and protein content of these fractions was determined using FM4-64 (AAT Bioquest, Sunnyvale, CA, USA) and Bradford assays, respectively, as previously described [27]. Samples of vesicle-containing fractions were precipitated with trichloroacetic acid (TCA) and analyzed by SDS-PAGE. Coomassie-stained bands were excised and subjected to tryptic digestion and ESI–MS analysis (IZKF Unit Core Proteomics, Münster, Germany).

### 2.7. Protein Labeling with Fluorescent Dyes

Labeling of AqdC and HQD*_S_._b_.* with the fluorophore DY™-488-NHS-Ester (Dyomics, Jena, Germany) was performed following the manufacturer’s instructions. Briefly, the *N*-hydroxysuccinimide (NHS) ester dye was dissolved in dimethylformamide (at 10 mg/mL), and a three-fold molar excess of the DY™-488-NHS-Ester solution was added to the proteins in a reaction buffer containing 50 mM NaHCO_3_ (pH 8.3). The labeling was developed for 2.5 h at 15 °C in the dark with continuous shaking. The unbound dye was removed from the protein–dye conjugates by dialysis against PBS (pH 7.4) using a 10 kDa Slide-A-Lyzer^®^ cassette (Thermo Scientific, Rockford, IL, USA) at 4 °C. The degree of labeling was determined spectrophotometrically as indicated by the manufacturer. The fluorescently labeled enzymes were aliquoted and stored at −20 °C until further use.

### 2.8. Homo-FRET-Based Interaction Assay

The interaction of dye-labeled AqdC and HQD*_S_._b_.* (5 µM) with *P. aeruginosa* extracellular membrane vesicles (MVs) was studied by taking advantage of the loss-of-polarization effect due to homo-FRET. This technique allows the detection of protein clusters in solution or in vivo by measuring the changes of anisotropy of a fluorescing sample [28]. The principle of measurement exploits the high degree of polarization that is commonly exhibited by a protein-bound fluorophore due to the slow rotation of the macromolecule [29]. When similarly labeled proteins cluster together, e.g., on the surface of a membrane, resonance energy transfer occurs between fluorophores, which leads to a depolarization of the emitted light. FP was measured using a Jasco FP-6500 spectrofluorometer equipped with polarization filters with excitation and emission wavelengths set at 488 nm and 519 nm at 10 nm bandwidth, respectively. The experiments were performed in PBS at room temperature. The absolute fluorescence polarization (*p*) of each measurement was calculated using Equation (1):

(1) $$p=\frac{I_{VV}-G\times I_{VH}}{I_{VV}+G\times I_{VH}}$$

where *I* denotes the fluorescence intensity, and the subscripts *V* and *H* represent, respectively, the vertical or horizontal position of the excitation (first subscript) or emission polarizer (second subscript); *G* denotes the instrument-dependent correction factor, which represents the instrument sensitivity ratio towards vertically and horizontally polarized light, according to Equation (2):

(2) $$G=\frac{I_{HV}}{I_{HH}}$$

## 3. Results and Discussion

The actinobacterial PQS dioxygenases AqdC as well as HQD*_S_._b_.* are quite instable at 37 °C, as reflected by a catalytic half-life (in PBS) of 0.49 h and 0.22 h, respectively [19]. When incubated in a 1:1 mixture of a 20 mM Tris-HCl buffer, pH 8, and sterile LB at 37 °C, AqdC and HQD*_S_._b_.* lost 52% and 79% of their activity, respectively, within 30 min [19]. Interestingly, culture supernatant of *P. aeruginosa* strain PA14 stabilized HQD*_S_._b_.*, as suggested by the observation that the enzyme retained 86% of activity when incubated for 30 min at 37 °C in a 1:1 mixture of buffer and supernatant of a stationary-phase PA14 culture grown in LB [19], raising the question of the causative agent(s). Remarkably, the stabilizing effect of the culture supernatant was not observed with any of the other PQS dioxygenases studied [19].

### 3.1. High-Molecular-Weight, Heat-Stable Factor(s) of P. aeruginosa Supernatant Stabilize(s) HQD_S_._b_.

**Figure 1:**
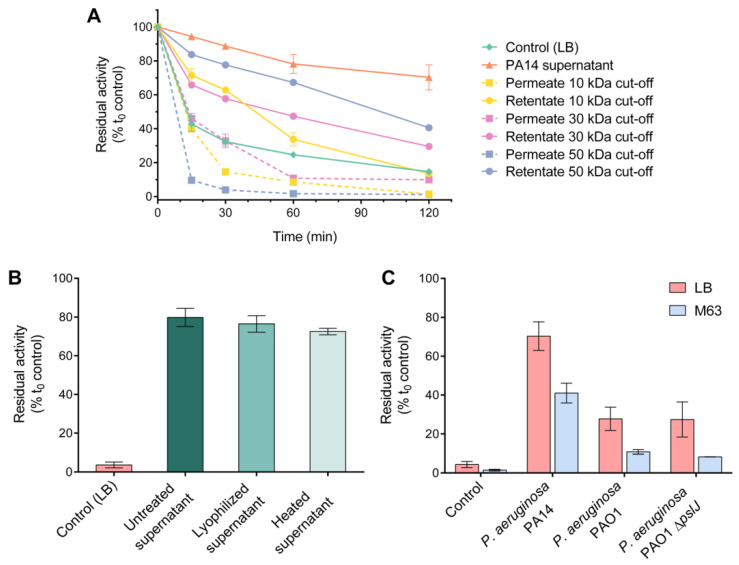
Residual enzymatic activity of HQD*_S_._b_.* upon incubation with (**A**) flow-through and retentate fractions of *P. aeruginosa* PA14 supernatant after ultrafiltration with 10 kDa, 30 kDa, 50 kDa cut-off membranes, (**B**) heat-treated (95 °C, 20 min), lyophilized, and untreated cell-free PA14 supernatant preparations and (**C**) supernatant fractions of *P. aeruginosa* PAO1, PAO1 ∆*pslJ* and PA14 strains, prepared in LB or M63 medium. In (**B**,**C**), the residual activity of HQD was determined after 120 min of incubation. All experiments were conducted at 37 °C. Sterile media were included as negative controls. Data represent the mean values ± SD of three replicates.

To roughly fractionate the supernatant components according to their molecular mass, the supernatant of *P. aeruginosa* PA14 cultures grown to stationary phase was subjected to ultrafiltration. Incubation of HQD*_S_._b_.* with the retentate fractions, especially those collected from filtration through a 50 kDa cut-off membrane, attenuated the decay in enzymatic activity compared to incubation in LB medium (Figure 1A). The permeate fractions did not stabilize the enzymatic activity, suggesting that stabilization was not due to an interaction with small molecules. Interestingly, heat treatment at 95 °C or lyophilization did not significantly affect the stabilizing effect of the PA14 supernatant (Figure 1B). The resistance of the stabilizing factor(s) to heat suggests that denaturation of supernatant proteins did not make a difference.

Culture supernatants of strain PAO1 also stabilized the enzyme, albeit to a lesser extent (Figure 1C). The effect of the supernatant of a *pslJ* mutant of strain PAO1, which is unable to produce the Psl exopolysaccharide [30] and in this respect mimics strain PA14, which due to an incomplete *psl* operon lacks Psl, did not differ from that of the wild-type PAO1 strain (Figure 1C), excluding this polysaccharide as being responsible for the different effects of the PAO1 and PA14 supernatants. Psl is produced by planktonic cells of strain PAO1 already in the exponential but mainly in the stationary phase and enables the cells to quickly attach to surfaces [31]. Irrespective of the *P. aeruginosa* strain tested, the residual activity of HQD*_S_._b_.* was considerably higher when a nutrient-rich medium (i.e., LB) was used for growth compared to the minimal medium M63

### 3.2. The HQD_S_._b_. Protein Shows Apparent Interactions with P. aeruginosa Extracellular Proteins

Potential interactions of HQD*_S_._b_.* with proteins or protein-containing particles present in the supernatant of strain PA14 were assessed in pull-down assays using His_8_-tagged HQD*_S_._b_.* as a bait. The mycobacterial PQS dioxygenase AqdC, whose activity was not altered when incubated with *P. aeruginosa* supernatant, was tested for comparison. An SDS-PAGE analysis of the pull-down eluates revealed that several prey proteins co-eluted with HQD*_S_._b_.*, whereas the co-eluate with AqdC contained hardly any proteins (Supplementary Figure S1).

Prominent proteins in the HQD*_S_._b_.* co-eluate were identified by MS analysis as GroEL (PA14_57010) and ArnA (PA14_18350). The 60 kDa chaperonin GroEL is a cytoplasmic protein, but is also abundant in the OMV proteome [32,33,34,35]. ArnA is a bifunctional UDP-glucuronic acid decarboxylase/UDP-4-amino-4-deoxy-l-arabinose formyltransferase that modifies lipid A with 4-amino-4-deoxy-l-arabinose. ArnA is predicted to be localized in the cytoplasm (*Pseudomonas* Genome Database, https://www.pseudomonas.com, accessed on 18 May 2022) [36], but was also identified among proteins associated with OMVs [32]. Additionally, peptides of ArnD (PA14_18340), a cytoplasmic deformylase also involved in lipid A modification, PanB (PA14_43830), a 3-methyl-2-oxobutanoate hydroxymethyltransferase involved in R-pantothenate biosynthesis predicted to be localized in the cytoplasmic membrane, and CcmE (PA14_45330), a cytochrome c biogenesis protein with an N-terminal signal peptide and a predicted transmembrane helix [37], were identified. CcmE has been previously identified in extracellular MVs from the human respiratory pathogen *Moraxella catarrhalis* [38]. While the molecular basis for the interaction of these *P. aeruginosa* proteins with HQD*_S_._b_.* in the pull-down approach remains elusive, their association with LPS precursors or with MVs prompted us to analyze the effect of extracellular vesicles and LPS on HQD*_S_._b_.*

### 3.3. HQD_S_._b_. Is Stabilized by P. aeruginosa Extracellular Membrane Vesicles

For the preparation of extracellular MVs, we followed the method of Bauman and Kuehn [26] and used MS analysis to verify the presence of major vesicle proteins in a TCA precipitate of the MV preparation. The precipitate contained the extracellular leucine aminopeptidase PaAP (PA14_26020) and the outer membrane porin proteins OprD (PA14_29220) and OprF (PA14_41570), consistent with the findings of Bauman and Kuehn that reported these proteins being amongst the six most abundant proteins in *P. aeruginosa* OMVs [26]. The MS analysis of our sample also revealed the alkaline protease AprA (PA14_48060) and the chitin binding protein CbpD (PA14_53250). Both are extracellular proteins which have previously been described within the *P. aeruginosa* OMV proteome [34,35].

**Figure 2:**
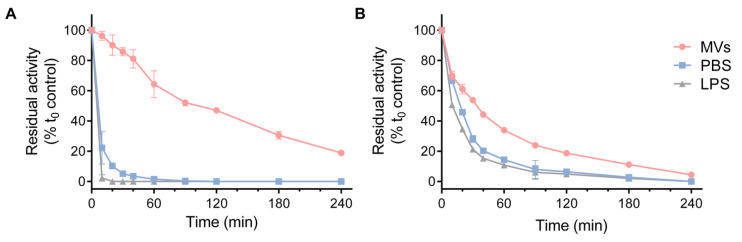
Stabilizing effect induced by the incubation of *P. aeruginosa* MVs with (**A**) HQD*_S_._b_*. and (**B**) AqdC. The enzymes were incubated at 37 °C with 0.2 mg/mL *P. aeruginosa* PA14 MVs (protein-based concentration), LPS (1 mg/mL in PBS) or PBS as a control. The residual enzymatic activity was determined at the specified time intervals. The data represent the mean ± SD of three replicates.

As illustrated in Figure 2A, the MV preparation from *P. aeruginosa* PA14 strongly attenuated the decrease in enzymatic activity observed when HQD*_S_._b_.* was incubated at 37 °C in PBS. The decay of enzymatic activity in the case of AqdC was more gradual in the presence of MVs than in PBS; however, the stabilizing effect was much less pronounced than that observed for HQD*_S_._b_.* (Figure 2B). Since LPS is a major constituent of OMVs, the effect of this component was also tested individually. However, commercially available *P. aeruginosa* LPS at 1 mg/mL (Figure 2), and also at lower concentrations tested, did not sustain the catalytic activity of the enzymes.

The observed resistance of the HQD*_S_._b_.*-stabilizing factor to lyophilization and heat (Figure 1B) is consistent with physicochemical properties reported for OMVs of different Gram-negative bacteria. Myxobacterial OMVs, for example, were shown to be stable to lyophilization [39] and to exhibit high tolerance to temperatures of up to 100 °C for several hours [40]. For OMVs from (mutant) *Salmonella* strains, incubation at 100 °C for 40 min did not affect particle size, lipid A penta-acylation and O-antigen-to-protein ratio, but protein denaturation was observed [41].

**Figure 3:**
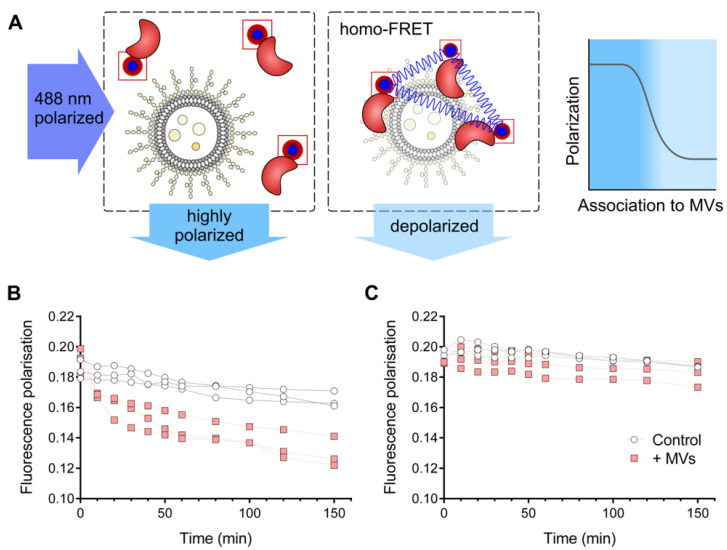
(**A**) Schematic illustration of the homo-FRET interaction assay with fluorescently labeled enzymes. The horizontal dark-blue arrow indicates the polarized excitation light. Interaction with or adsorption of the fluorescently labeled enzyme (red) to the vesicle surface brings the fluorophore molecules (blue) into spatial proximity, leading to FRET and hence to a decrease in polarization, indicated by the vertical light blue arrow (homo-FRET box, center). Without interaction with MVs, the emitted light (vertical blue arrow) remains highly polarized (left box). (**B**,**C**) Fluorescence polarization of (**B**) labeled HQD*_S_._b_.*-Dy488 and of (**C**) AqdC-Dy488 in the presence or absence (control) of 0.2 mg/mL of *P. aeruginosa* MVs (protein-based concentration), measured at 488 nm excitation and 519 nm emission wavelength over 150 min at the indicated time intervals. Three independent experiments were performed. The lines connecting the data points collected from each replicate are for illustrative purposes only.

To characterize the effect of the *P. aeruginosa* vesicle fraction further, we tested whether interaction with or adsorption to the vesicle surface would play a role for stabilizing the protein. The physical interaction of HQD*_S_._b_.* with the MVs was probed by an assay in which spatial clustering of the protein was detected by homo-FRET of fluorescently labeled HQD*_S_._b_.* molecules. This assay has the advantage of requiring a minimal labeling effort, which moreover did not affect the catalytic activity of the enzyme. The adsorption of fluorescently labeled proteins onto, e.g., a liposome surface brings individual proteins into spatial proximity, which leads to the engagement of fluorophores in radiation-free energy transfer (Figure 3A). This effect, referred to as homo-FRET, does not alter the observed spectral characteristics but leads to a decrease in fluorescence polarization, which can be used as readout of clustering. Addition of the MV preparation to dye-labeled HQD*_S_._b_.* indeed resulted in a decrease of fluorescence polarization (Figure 3B), while no significant change in the fluorescence polarization levels was observed when the same experiment was performed with AqdC (Figure 3C).

It has to be noted that, in general, fluorescence polarization depends on several factors. While temperature, solvent and physicochemical properties of the dye were constant between the experiments, the labeling positions in AqdC and HQD*_S_._b_.* varied due to the dissimilarity of their amino acid sequences. This could lead to different tendencies to double labeling (two highly accessible lysine residues may lead to increased double labeling). Different rates of double labeling, as well as different tendencies of the generally monomeric enzymes to form dimers or even trimers [42], cause different rates of “intrinsic” homo-FRET, which can explain the slight variation observed for AqdC and HQD*_S_._b_.* at the start of the experiments. However, for a strong gradual decrease in polarization as observed in Figure 3A for HQD*_S_._b_.* in the presence of MVs, a clustering of many fluorophores in the FRET-relevant range below 10 nm has to occur [43,44], which can only be explained by the progressive accumulation of HQD*_S_._b_.* in solution. We can exclude the gradual aggregation of HQD*_S_._b_.* due to precipitation, as this would have led to a decrease of the total fluorescence intensity during the experiment, which was not observed, confirming that the accumulation of HQD*_S_._b_.* was mediated by the interaction with the MVs.

Interestingly, studies by Florez et al. [45] revealed that the *P. aeruginosa* strain PA14 is a stronger OMV producer than the strain PAO1; however, PA14 MVs appear to be smaller than those from strain PAO1 [46]. These properties might contribute to the differences in residual activities observed when HQD*_S_._b_.* was incubated in the presence of PA14 supernatant compared to PAO1 supernatant (Figure 1C).

Since HQD*_S_._b_.* is presumed to be a cytoplasmic protein, it is difficult to draw conclusions on the molecular basis of its adsorption to MVs. However, many cytoplasmic proteins lacking an identifiable membrane anchor are peripherally and reversibly associated with the inner membrane via mostly electrostatic and moderate hydrophobic interactions, as studied in most detail for *Escherichia coli* [47,48]. In the case of HQD*_S_._b_.*, weak interactions with surface polysaccharides or with membrane surface charges might suffice for the observed adsorption. Notably, commercially available purified LPS failed to stabilize the enzymatic activity, tentatively suggesting that the ordered LPS-phospholipid bilayer of the vesicles is important for the interaction and/or stabilization.

To get an idea of how HQD*_S_._b_.* could facilitate an OMV interaction, we analyzed the structure of AqdC and a homolog (crystal structures 6RA2, 2WJ3) as well as several AqdC homology models, including that of HQD*_S_._b_.* (models generated with Alphafold 2 [49,50]), the results of which were analyzed for surface charge distribution as calculated by an adaptive Poisson–Boltzmann solver. Since *P. aeruginosa* OM surfaces as well as secreted OMVs are charged negatively [51,52], we scanned for positive patches of charge that could induce their interaction and stand out in HQD*_S_._b_.* compared to other structures. A visual inspection revealed that the surface charge distribution is highly conserved among AqdC homologs, with no unique positive patches identifiable in HQD*_S_._b_.* (Supplementary Figure S2). The mechanism of the observed binding to extracellular MVs thus remains elusive.

From a bacteriological perspective, binding of a PQS-cleaving enzyme to membrane surfaces is highly reconcilable with its physiological function, since the hydrophobic substrate PQS also accumulates in membranes, preferentially interacting with LPS in the outer membrane [53]. Lowering the diffusion barrier through (outer) membrane association of the enzyme therefore increases its catalytic efficiency. Considering that HQD*_S_._b_.* is an intracellular enzyme of *S. bingchenggensis*, its interaction with OMVs from *P. aeruginosa* at first glance seems unlikely. However, cell death, which may lead to the release of the cytoplasmic enzyme, is part of the streptomycetal developmental cycle. Moreover, *Streptomyces* spp. also shed extracellular vesicles, and as shown for the model organism *S. coelicolor*, their cargo contains numerous proteins, the majority of which were annotated as cytoplasmic (61%) or peripheral membrane proteins facing the cytoplasm (29%) [54]. In soil habitats, an encounter of HQD*_S_._b_*., released by autolysis or programmed hyphal death from *S. bingchenggensis,* or released from extracellular vesicles of *S. bingchenggensis*, with *P. aeruginosa* OMVs would likely allow the efficient quenching of the PQS signal molecule.

## Data Availability

The data presented in this study are available in the Figures of the article and the Supplementary Materials. Strains are available upon request from the corresponding author.

## Supplementary Materials

The following supporting information can be downloaded at: https://www.mdpi.com/article/10.3390/biom12111656/s1, Figure S1. Apparent interaction of the PQS dioxygenases with extracellular proteins of *Pseudomonas aeruginosa* PA14, indicated by co-elution of (A) HQD*_S_._b_.* and (B) AqdC with proteins from *P. aeruginosa* PA14 culture supernatants. Figure S2. Scheme of the electrostatic surface potential of the dioxygenases HOD, AqdC, AqdC2, HQD*_N_._f_.* and HQD*_S_._b_.* References [49,50] are cited in the supplementary materials.
