# Supplementary material for: A PQS-Cleaving Quorum Quenching Enzyme Targets Extracellular Membrane Vesicles of Pseudomonas aeruginosa
Source: Biomolecules. 2022 Nov 8;12(11):1656. doi: 10.3390/biom12111656 (PMC9687290; doi:10.3390/biom12111656)
Supplement: Supplementary file 1 [file biomolecules-12-01656-s001.zip › biomolecules-1992770-supplementary-conversion.pdf]

Supplementary material to:

A PQS-Cleaving Quorum Quenching Enzyme Targets Extracellular Membrane Vesicles of Pseudomonas aeruginosa

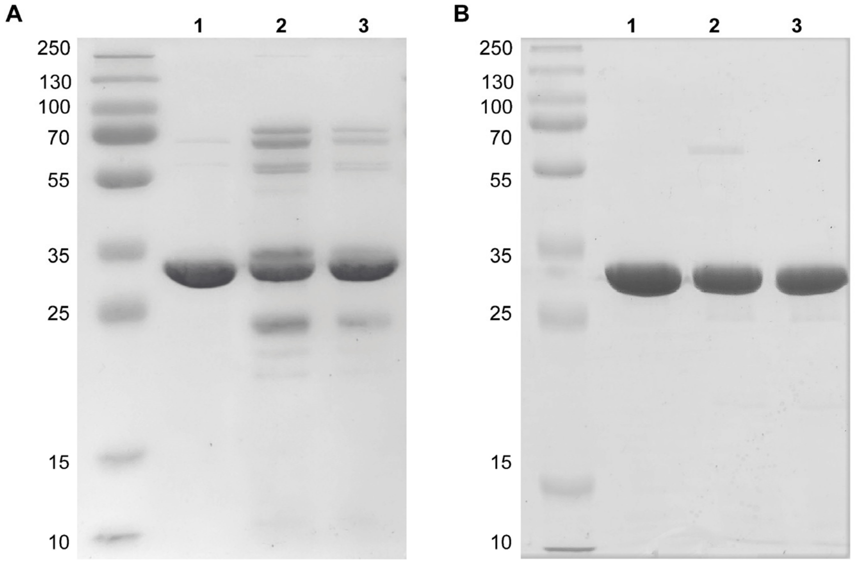

Figure S1. Apparent interaction of the PQS dioxygenases with extracellular proteins of *Pseudomonas aeruginosa* PA14, indicated by co-elution of (**A**) $HQD_{S.b.}$ and (**B**) AqdC with proteins from *P. aeruginosa* PA14 culture supernatant. 12.5% SDS-PAGE (Coomassie-stained). Lanes 1: $HQD_{S.b.}$ (A) and AqdC (B) purified by Ni^{2+}-NTA affinity chromatography; lanes 2: $HQD_{S.b.}$(A) and AqdC (B) were incubated for 1 hour with PA14 supernatant and subsequently subjected to Ni^{2+}-NTA affinity chromatography; lanes 3: $HQD_{S.b.}$ (A) and AqdC (B) were loaded to the Ni^{2+}-NTA column and washed with PA14 supernatant prior to elution. PageRuler™ Plus Prestained Protein Ladder (Thermo Scientific) was used as marker with molecular masses provided in kDa.

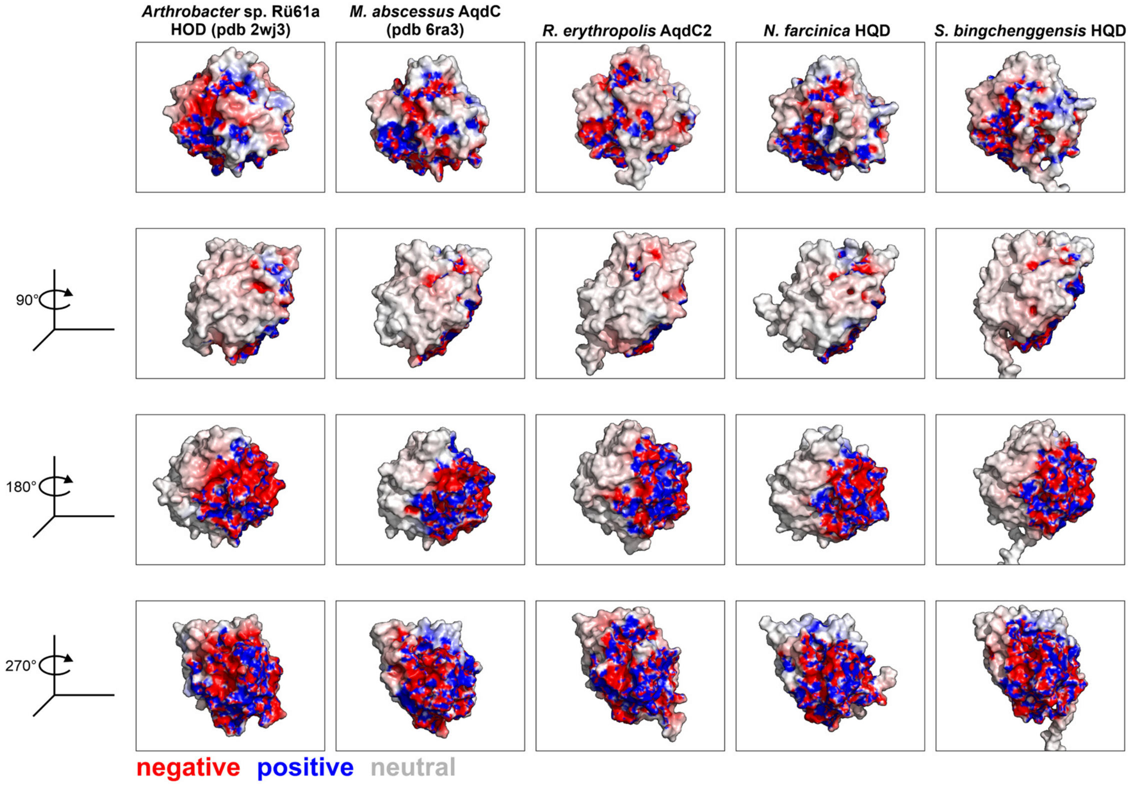

Figure S2. Scheme of the electrostatic surface potential of the dioxygenases HOD, AqdC, AqdC2, $HQD_{N.f.}$ and $HQD_{S.b.}$. Structural models of the enzymes were generated with AlphaFold [49,50] and their electrostatic properties were calculated using the Poisson-Boltzmann equation. Blue and red represent positive and negative electrostatic surface potential, respectively, whereas white color indicates neutral electrostatic surface potential.
